# Different Models of Ophthalmology Care for People Experiencing Homelessness

**DOI:** 10.3390/medicina61122178

**Published:** 2025-12-08

**Authors:** Caroline Campbell, Anindya Samanta, Catherine Reppa, Jay Chhablani

**Affiliations:** 1 School of Medicine, Texas Tech University Health Sciences Center, Lubbock, TX 79430, USA; caroline.campbell@ttuhsc.edu; 2 Department of Ophthalmology & Visual Sciences, Texas Tech University Health Sciences Center, Lubbock, TX 79430, USA; catherine.reppa@ttuhsc.edu; 3 Southwest Retina Consultants, PA, 7100 Curie Drive, 3800, El Paso, TX 79902, USA; 4 Department of Ophthalmology, University of Pittsburgh, 203 Lothrop Street, Pittsburgh, PA 15213, USA

**Keywords:** homeless ophthalmology care, persons experiencing homelessness ophthalmology

## Abstract

*Background and Objectives*: People experiencing homelessness (PEH) face a disproportionately high burden of vision impairment, most commonly from uncorrected refractive error (RE), and encounter significant barriers to accessing care. Despite these challenges, there is limited knowledge about effective approaches to providing ophthalmic services to this population. This review aims to categorize and evaluate existing models of eye care delivery for PEH in North America. *Materials and Methods*: A literature search was conducted for publications between 2013 and 2023. Eligible studies included those describing direct ophthalmic interventions for PEH in North America. Identified studies were reviewed and classified into distinct models of care delivery. *Results*: Four models of care emerged: office-based, shelter-based, mobile/temporary-based, and street medicine-based. Each model demonstrated unique strengths and limitations related to accessibility, continuity of care, and resource intensity. Across models, on-site correction of RE, particularly through provision of eyeglasses at the point of care, led to documented improvement of vision. However, referral completion and follow-up rates to tertiary care centers were low, especially in programs where services were fragmented across multiple locations. Strategies that emphasize same-location diagnosis and treatment for RE increase service delivery rates. Further studies are needed to evaluate referral pathways, long-term outcomes, and policy strategies to reduce vision-related disparities in this underserved population. *Conclusions*: No single model of care proved universally superior. Instead, hybrid approaches that integrate multiple models tailored to community infrastructure and patient needs appear most effective for expanding access to ophthalmic services among PEH.

## 1. Introduction

Among people experiencing homelessness (PEH), vision impairment is a pressing issue and is independently related to other unmet needs for mental health and dental care [1]. Having poor vision can also be a barrier to navigating the healthcare system for PEH [2]. Additionally, a common cause of impaired vision among PEH is uncorrected refractive error (RE), followed by cataracts, corneal and external eye disease, glaucoma and retinal disease [3]. According to a meta-analysis by Sayal et al. (2021), 25% of PEH had non-RE ocular pathology [3]. A retrospective study comparing PEH at a free clinic to housed patients at a tertiary center found that PEH were more likely to have worse visual acuity and higher rates of vision-threatening conditions, including more severe diabetic retinopathy, glaucoma, and cataracts. They were more likely to be referred for follow-up or surgery compared to their housed counterparts [4].

However, many PEH face significant barriers to ophthalmic care, including transportation difficulties, lack of insurance, limited awareness of available resources, and negative prior experiences with healthcare providers [5,6,7,8]. Several models have been developed to overcome these barriers and deliver ophthalmic services.

## 2. Methods

For this narrative review, a literature search was performed on 15 December 2023, using Embase, Google Scholar, and MEDLINE. CC and AS conducted a search for ophthalmic care for PEH using the terms “eye care” or “ophthalmology” and “homeless,” “unhoused,” or “unsheltered.” Publications from 1 January 2013 to 15 December 2023 were included. Eligible studies focused on PEH and also included an intervention, such as distribution of glasses or referral to a higher level of care. Studies that reported only demographic data, pathology, or involved a one-time questionnaire were excluded. Studies conducted outside North America were excluded to ensure consistency in health system and populations.

## 3. Results

A total of 14 articles were identified. CR confirmed the articles were consistent with the inclusion and exclusion criteria (Table 1).

The studies highlighted a wide range of interventions aimed at improving eye care among PEH. Previously, these studies were briefly discussed, but a consistent framework is needed to describe how this care is delivered [9]. Rather than evaluating each study for its quality or outcomes, this narrative review aimed to categorize the models of care and define key characteristics, advantages, and disadvantages of each model. Four distinct models of ophthalmic care were identified: office-based, shelter-based, mobile/temporary-based, and street medicine-based (Table 2) [10]. Each model has distinct advantages and limitations (Table 3).

**Table 1:** Articles identified through the literature search, organized according to the four models of ophthalmology care for people experiencing homelessness (PEH): office-based, shelter-based, mobile/temporary, and street medicine. OTC: over the counter; N/A: not available; * the data was presented as a figure.

Model	Name	Year	Sample Size	Services	On-Site vs. Off-Site Dispensing	Spectacle Fulfillment (n/N)	Referral Rate (n/N)	Follow-Up (n/N)	Surgery
Office-based	Pine Street Inn [11]	2021	424	Eye exam, refraction ± perimetry and gonioscopy	On-site OTC reading glasses, offsite prescription glasses	274/356	61/424	20/61	N/A
	Kansas City Free Eye Clinic [12]	2018	334	Eye exams and refraction	N/A	N/A	N/A	N/A	N/A
Shelter-based	Kansas City Free Eye Clinic [13]	2023	384	Eye exams and refraction	On-site	~350 *	N/A	2 visits—84/384 3 visits—14/384 4 visits—3/384 5 visits 1/384	N/A
	UCSF Ophthalmology Shelter Clinic [14,15,16]	2021	68	Eye exams and refraction	Off-site through non-profit organization	14/40	17/68	7/17	N/A
		2021	71	Eye exams and refraction	Off-site through non-profit organization	28/71	14/71	6/14	N/A
		2020	123	Eye exam and refraction	Off-site through non-profit organization	N/A	N/A	N/A	N/A
	Georgetown Eye Health Initiative [17]	2023	N/A	Eye exams and refraction	N/A	N/A	N/A	N/A	N/A
Mobile/temporary-based	Regard Collectif [18]	2018	308	Eye exam and refraction	N/A	179/308	N/A	N/A	N/A
	Homeless shelters [19,20,21,22]	2015	100	Undilated fundoscopy	N/A	N/A	32/100	N/A	N/A
		2018	86 children, 55 adults	Eye exams and refraction	N/A	N/A	15/86 children, 23/55 adults	N/A	N/A
		2019	143	Eye exams and refraction	N/A	N/A	43/143	N/A	N/A
		2022	93	Eye exams and refraction	N/A	N/A	N/A	38/93	4
	Outdoor Tent [23]	2023	11	Eye exams and refraction	On-site OTC reading glasses	N/A	2/11	N/A	N/A
Street medicine-based	Allegheny Health Network [24]	2023	117	Autorefraction	On-site	80/88	23/117	61/117	1 cataract surgery

**Table 2:** Description, infrastructure, and primary target population of each model of ophthalmologic care for people experiencing homelessness (PEH).

Types of Clinics	Description	Infrastructure	Primary Target
Office-based	Brick-and-mortar outpatient eye clinics	Permanent	PEH and housed, underserved patients
Shelter-based	Clinic located inside or adjacent to a homeless shelter	Permanent	Shelter residents
Mobile/temporary-based	Portable clinics, mobile vans, or event-based setups	Temporary	PEH in shelters or in the community
Street medicine-based	Outreach care delivered with portable equipment in unsheltered settings	Permanent	Unhoused PEH

**Table 3:** Advantages and limitations of each model of ophthalmologic care for people experiencing homelessness (PEH).

Types of Clinics	Advantages	Limitations
Office-based	Stable location supports continuity of care Regular operating hours improve access Shared location with primary care, dental, mental health services Ability to deliver long-term follow-up	Transportation barriers to fixed site May be intimidating due to formal medical environment Appointment systems require phone access, difficult for PEH to communicate
Shelter-based	Improved trust due to familiar setting and personnel Integrated with shelter services resulting in better coordination, proactive referrals Potential for continuity while patient resides in shelter	Access limited to shelter residents; difficult for new patients High shelter turnover disrupts continuity and follow-up lost if patient leaves shelter
Mobile/temporary-based	High outreach potential; visible and accessible Flexibility to bring care directly to PEH across the city Reduces transportation barriers	Limited duration Long-term care in a second location Weather constraints for outdoor events Repeated setup/teardown is labor-intensive
Street medicine-based	Reaches unsheltered PEH who avoid shelters Eliminates major access barriers including transportation Builds on strong existing provider–patient relationships	Requires well-developed street medicine infrastructure Trust-building is time- and personnel-intensive Limited diagnostic capability compared to clinics

## 4. Office-Based Model

In the office-based model, PEH receive eye care at brick-and-mortar clinics. These clinics may serve a mixed population that includes underserved, housed patients, or be exclusively dedicated to PEH. These clinics are often either part of the federal healthcare for the Homeless (HCH) program or a free clinic associated with an academic center [7,11,12]. Patients are either referred to by their primary care physician or are self-referred to the clinic [11]. One of the best-documented programs is the Boston HCH Program, which incorporates ophthalmic care through its Pine Street Inn Eye Clinic. Over a period of one year, this clinic provided comprehensive eye exams, including slit lamp biomicroscopy and dilated fundus exams, to a total of 424 patients. A comparable program at Baltimore HCH similarly examined patients and referred if necessary to a higher level of care [7].

An example of an office-based clinic location that serves both PEH and housed patients is the Kansas City Free Eye Clinic (KCFEC). Over a period of four years, the clinic saw 334 patients, of which 57.78% self-reported as PEH [12]. The clinic, at the time, was located near multiple shelters and allowed both appointments and several walk-in slots.

One advantage of this model is that patients have a fixed location to follow-up and continue care. These clinics typically offer regular operating hours, improving accessibility and scheduling. Due to their permanent infrastructure, these clinics are well-equipped to provide long-term care over an extended period of time [25]. Eye clinics are usually part of multi-specialty clinics located either in the same location or same health system, allowing PEH to also gain access to primary healthcare, dental services, well-woman care, wound services, and mental health [26].

A key limitation is the fixed location of these clinics, which may present transportation barriers for PEH. PEH may have perceived barriers to access including being intimidated by a formal clinic setting or previous experiences involving discrimination and dehumanization [27,28]. Additionally, the inflexible appointment-based system may limit access and make it difficult for PEH to navigate due to lack of access to a cellphone or electronic device [5,27].

## 5. Shelter-Based Model

The second approach is a permanent clinic that is located within or adjacent to a homeless shelter. Only a minority of clinics are located inside a shelter, a number that is even smaller for eye clinics [13]. Along with providing services such as food, shower facilities, laundry services, and case management, these shelters can also provide medical services via on-site clinics [13]. The target of the clinic is the residents residing in the shelter. These clinics typically occur every few weeks and are usually staffed by faculty, residents, and students from a nearby academic center [13,14,15,16,17,29]. For example, over a period of 4 years, the KC Free Eye Clinic with a location in a homeless shelter, saw 384 patients, with over 82% seen in the shelter setting. 102/384 (26.5%) of patients had at least two visits during that period [13].

An advantage of this model is the greater potential for continuity of care due to access of multiple resources in a timely and consistent manner [13]. As there is integration with other services and staff in the shelter, there may be greater trust and rapport. This may also lead to proactive identification of health needs and referrals from the shelter itself [13]. Patients may also feel more comfortable seeking care in an already familiar environment.

However, a disadvantage is that the scope of these clinics is often exclusively established for PEH in the shelter. Therefore, it is difficult for new patients to receive treatment in these facilities. Due to the high turnover of the residents at the shelter, it can be difficult to establish continuity and trust between the staff and the patients. Continuity of care may also be disrupted if patients leave the shelter before follow-up is completed.

## 6. Mobile/Temporary-Based Model

The mobile/temporary-based model involves establishing short-term clinics that operate for a limited duration. A defining feature of this model is the use of portable equipment, allowing for flexible deployment across a variety of settings. These clinics are more likely to be one-time events; they can be located in homeless shelters, homeless encampments, or community areas.

One common example of this model is the use of a mobile van equipped with ophthalmic equipment to set up a temporary day clinic. For example, this model was implemented by the University of Montreal School of Optometry. By operating a mobile clinic to visit shelters three to four times per month, PEH (n = 308) were screened over the course of a year [18]. Other studies have also reported temporary clinics operating within shelters [19,20,21,22].

A subset of the temporary-based model is the event-based model. Unlike mobile vans, stationary clinics are set up at fixed locations to maximize outreach. For example, three outdoor clinics were held at a downtown park in Toronto, screening PEH (n = 11) [23]. A portable tent was used to store and house the equipment. Another example is the community health fairs, which are held periodically and provide free vision screenings for both underserved individuals and PEH in the community.

Advantages of this model include high visibility and strong outreach potential, particularly when events are well-publicized and organized in partnership with community organizations. This facilitates opportunistic engagement, attracting individuals who may not otherwise seek care. The model also provides flexibility regarding location, helping to overcome transportation barriers by bringing services directly to PEH in different parts of the city.

However, limitations include the short duration of service availability, which can restrict access to follow-up care. Since long-term care must be performed at a different location, follow-up rates may be low. Weather conditions and temperature can pose challenges to outdoor events. Additionally, repeated setup, breakdown, and transportation of equipment can be logistically burdensome, potentially reducing efficiency.

## 7. Street Medicine-Based Model

Street medicine delivers healthcare services to PEH, particularly those who are unsheltered and reside in locations such as encampments, parks, and under bridges [30]. Although the street medicine model is relatively new in the field of ophthalmology, it has shown promise in addressing vision-related needs of this population. The street medicine team at Allegheny Health Network (AHN) in Pittsburgh, PA, implemented an ophthalmology-designed protocol to treat RE and screen for non-RE pathology [31]. Using a handheld vision chart, pinhole glasses, and a portable auto-refractor, the team successfully identified RE. Other street medicine teams have also conducted ophthalmological screenings in non-ambulatory settings, such as public transportation hubs, for both PEH and underserved populations [32].

This model targets a particularly vulnerable subset of PEH, those who actively avoid shelters and rely heavily on their vision for daily navigation and safety [31]. Key advantages to this approach include its ability to eliminate common barriers to care cited by PEH, such as a lack of transportation, absence of secure storage for belongings, and the inability to leave a companion unattended [33,34]. Additionally, the use of portable equipment and integration into existing street medicine infrastructure creates a low barrier of entry for care. Existing patient–provider relationships within street medicine teams may also help reduce stigma and increase acceptance of care. As a result, PEH were generally more receptive to visual screening or treatment. Positive clinical experiences also encouraged patients to open a dialogue regarding non-ocular health concerns [9].

One limitation of this model is its reliance on portable equipment and the need for coordination within an established street medicine network. Scalability of street medicine remains difficult due to inadequate primary care reimbursements and the need to heavily rely on charitable funding [33]. Additionally, the model relies on building patient trust, a process that is difficult without time-consuming foundational work laid by social workers and care coordinators.

## 8. Treating Refractive Error

As previously stated, RE is the most prevalent ocular issue among PEH [3,7]. Treating RE is the most cost-effective ocular intervention for improving vision and should be prioritized in this population [7,35]. A previous study demonstrated an improvement of up to five lines in visual acuity following the distribution of glasses and treatment of RE [9]. Correcting RE in a non-ambulatory setting facilitates the identification of non-RE, which can be referred for further evaluation at a tertiary center [9].

All care models were shown to improve access to ophthalmic services for PEH, which historically has been difficult. RE is treated through the distribution of free reading glasses, referrals for prescription glasses, or obtaining prescription glasses. A key trend across the studies is that glasses distribution is more successful when patients receive glasses in the same location as their eye examination, regardless of the care model.

In an office-based care model, the Pine Street Clinic distributed glasses to 77% of patients, either by providing readers during the initial visit or by ordering prescription lenses [11]. 71% of the PEH prescribed glasses successfully received them. The AHN street medicine team achieved a 90.1% glasses delivery success rate using a combination of on-site delivery and designated drop-in center pickups [9]. Similarly, KCFEC had patients pick up eyeglasses in the same location in 1–2 weeks [13]. This study did not report specific numbers; it did note that the reliable location of the eye clinic provided enhanced access for PEH in obtaining new glasses.

In contrast, programs requiring patients to travel to a separate site to collect glasses demonstrated a substantially lower distribution rate. The shelter-based care clinic model at UCSF referred patients to an external nonprofit organization for free glasses, but only 35% of PEH obtained their free glasses over the course of one year [14].

These findings reinforce the principle that RE should ideally be both diagnosed and treated during the same clinical encounter, at the same physical location, to maximize treatment adherence and impact.

## 9. Referral and Follow-Up Rates

Follow-up rates to tertiary ophthalmology clinics among PEH were variable but generally on the lower end. Compared to their housed counterparts, PEH were significantly less likely to follow-up, despite having a higher disease burden [4]. In a retrospective study comparing 170 PEH at a free homeless clinic to 181 patients at a tertiary referral center, only 59% of the PEH completed their surgery, compared to 100% of the housed patients.

In an office-based model, the Pine Street Clinic had 14% referral rate to the tertiary clinic, but only 32.0% of referred patients followed up, despite 98% having insurance coverage [11]. In the AHN street medicine study, 19.7% of patients were referred for further evaluation of non-RE pathology; 40.1% of the 23 patients completed their appointment, including surgical intervention [24]. At the UCSF shelter-based clinic, 17/68 (25%) of patients were referred, with 58.8% completing their follow-up appointment [14].

Other studies reported referral rates but did not disclose the number of completed follow-ups. Over a two-year period, 46% of the 162 PEH were referred to a tertiary eye clinic through Baltimore HCH [5]. For event-based models, 12/124 (9.7%) and 43/143 (30.0%) PEH were referred to a tertiary clinic by the Project Vision in Hawaii and temporary screenings performed in homeless shelters in Canada, respectively [8,20]. In one event-based temporary clinic, 2/11 (18.2%) were referred for ophthalmology care, and one successfully underwent cataract surgery [23].

Similar to patterns seen in glasses distribution, a common trend is that PEH are less likely to follow through when care requires visiting a second location for follow-up. Therefore, eye care should be provided at the same location whenever possible to maximize treatment impact during each encounter. When necessary to follow up in a tertiary center, health coaching and assisting with transportation are strategies to improve follow-up rates [14]. Greater emphasis needs to be placed on tracking patients, to provide more insights into identifying factors that lead to greater follow-up rates.

As follow-up rates are often suboptimal, obtaining more comprehensive diagnostic information during the initial visit may reduce the total number of follow-up appointments required. Incorporating portable auto-refractors, Optical Coherence Tomography, and fundus cameras may help in greater diagnostic information during the initial visit [36,37,38]. Another potential approach involves telehealth visits conducted within physical locations, where trained non-physicians can obtain auto-refraction, conduct diagnostic testing and/or conduct an examination, so the physician can determine management and follow-up [39].

Many organizations employ multiple models concurrently to deliver care and meet the diverse need of PEH in their communities. For example, the federally qualified HCH in Houston, Texas has three stand-alone office-based clinics, six shelter-based clinics, a mobile dental unit, and a street medicine team [26,40]. While vision care at HCH Houston is primarily performed through their office-based clinic, other groups have adopted blended care models to serve the ophthalmic needs of their local PEH population [12,13,19,25].

## 10. Limitations

There are limited studies published on eye care for PEH. While this paper highlights recent work focused on primary screening in this population, evidence on subsequent care such as referral success rates and follow-up in tertiary setting remains scarce. Further research is needed to inform effective policies on eye care delivery for PEH in North America.

## 11. Conclusions

Each care model described has distinct advantages and limitations. No single model has emerged to be superior. Instead, the choice of delivery should be guided by the local needs, available resources, and specific needs of the target population. Increasing awareness of the strengths and adaptability of various ophthalmic care models may support broader implementations and enhanced access across a range of clinical and community services.

## Data Availability

No new data were created or analyzed in this study.
